# Sex and Rate Change Differences in QT/RR Hysteresis in Healthy Subjects

**DOI:** 10.3389/fphys.2021.814542

**Published:** 2022-02-07

**Authors:** Irena Andršová, Katerina Hnatkova, Martina Šišáková, Ondřej Toman, Peter Smetana, Katharina M. Huster, Petra Barthel, Tomáš Novotný, Georg Schmidt, Marek Malik

**Affiliations:** ^1^Faculty of Medicine, Department of Internal Medicine and Cardiology, University Hospital Brno, Masaryk University, Brno, Czechia; ^2^National Heart and Lung Institute, Imperial College, London, United Kingdom; ^3^Wilhelminenspital der Stadt Wien, Vienna, Austria; ^4^Klinikum rechts der Isar, Technische Universität München, Munich, Germany; ^5^Faculty of Medicine, Department of Internal Medicine and Cardiology, Masaryk University, Brno, Czechia

**Keywords:** QT/RR adaptation, QT/RR hysteresis, healthy subjects, non-linear regression modelling, best-fit models, sex differences, age influence

## Abstract

While it is now well-understood that the extent of QT interval changes due to underlying heart rate differences (i.e., the QT/RR adaptation) needs to be distinguished from the speed with which the QT interval reacts to heart rate changes (i.e., the so-called QT/RR hysteresis), gaps still exist in the physiologic understanding of QT/RR hysteresis processes. This study was designed to address the questions of whether the speed of QT adaptation to heart rate changes is driven by time or by number of cardiac cycles; whether QT interval adaptation speed is the same when heart rate accelerates and decelerates; and whether the characteristics of QT/RR hysteresis are related to age and sex. The study evaluated 897,570 measurements of QT intervals together with their 5-min histories of preceding RR intervals, all recorded in 751 healthy volunteers (336 females) aged 34.3 ± 9.5 years. Three different QT/RR adaptation models were combined with exponential decay models that distinguished time-based and interval-based QT/RR hysteresis. In each subject and for each modelling combination, a best-fit combination of modelling parameters was obtained by seeking minimal regression residuals. The results showed that the response of QT/RR hysteresis appears to be driven by absolute time rather than by the number of cardiac cycles. The speed of QT/RR hysteresis was found decreasing with increasing age whilst the duration of individually rate corrected QTc interval was found increasing with increasing age. Contrary to the longer QTc intervals, QT/RR hysteresis speed was faster in females. QT/RR hysteresis differences between heart rate acceleration and deceleration were not found to be physiologically systematic (i.e., they differed among different healthy subjects), but on average, QT/RR hysteresis speed was found slower after heart rate acceleration than after rate deceleration.

## Introduction

The dependency of QT interval duration on underlying heart rate has been known practically since the beginning of clinical electrocardiography (Waller, 1887; Einthoven, 1903). This dependency (the slower the heart rate, the longer the QT interval) also reflects even older knowledge of rate influence on the duration of mechanical systole, which was assessed by auscultation and mechanical apexograms well before the first recordings of electrical manifestation of cardiac activity. Indeed, in 1870, Garrod already suggested that the duration of systole changes with the cube root of the cardiac period (Garrod, 1870). Later, when using a mechanical cardiograph rather than a sphygmograph, Garrod proposed that the duration of systole relates to square root of the cardiac cycle (Garrod, 1875). This all happened almost half a century before corresponding proposals have been made concerning electrocardiographic QT interval measurements (Bazett, 1920; Fridericia, 1920).

**Figure 1:**
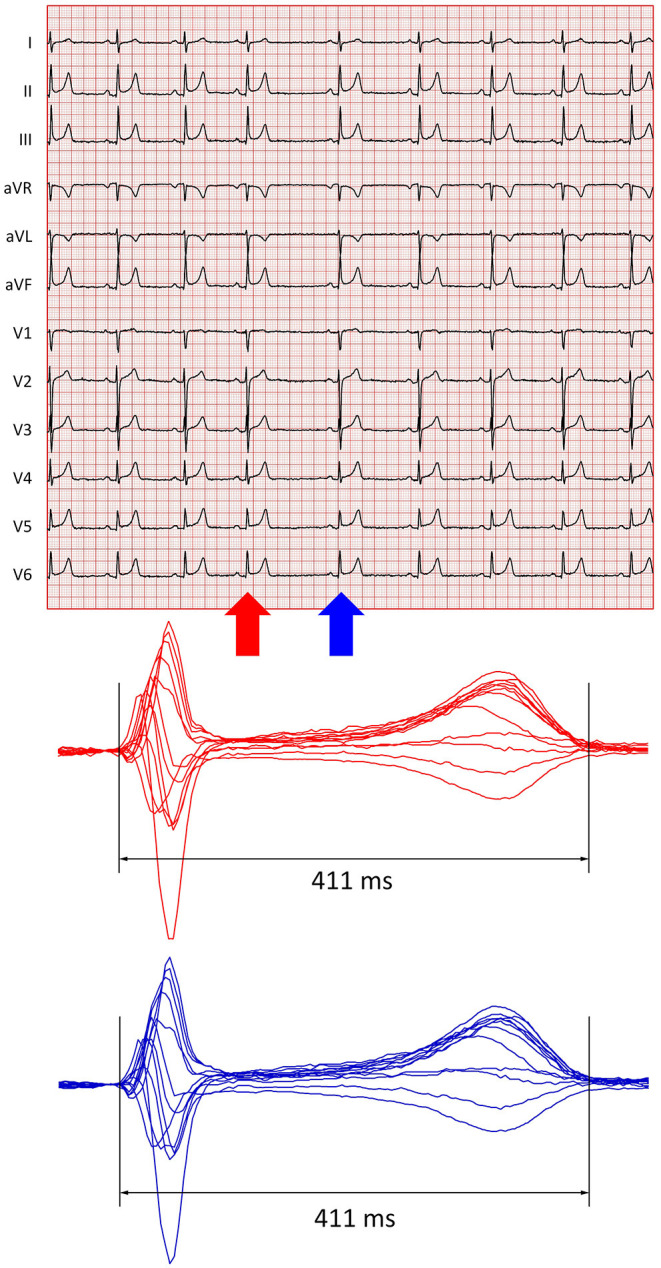
Example of short-term QT/RR relationship. The top panel shows a 10-s electrocardiogram of a 22-year-old healthy female. The recording shows substantial sinus arrhythmia. The shortest and longest RR intervals were 1,019 and 1,528 ms, respectively. The red and blue arrows mark QRS complexes preceded by the shortest and longest RR intervals, respectively. The bottom panel shows images of superimposed QRS-T complexes of all the 12 leads of these complexes (in the respective colours). The QT interval measurements ensuring morphological correspondence (Hnatkova et al., 2009) of both complexes led to the same QT interval of 411 ms. Thus, relating each of the QT intervals of the tracing to the preceding RR interval (or assuming a substantial immediate beat-to-beat effect of each RR interval on the subsequent QT interval) would have led to substantial inaccuracy (for instance, if these two QT interval measurements are corrected for the preceding RR interval using the Fridericia Formula, a physiologically non-sensical beat-to-beat QTc change of more than 50 ms is calculated).

During the subsequent century, however, much lesser, and much more recent attention has been given to the dynamics of the QT/heart-rate dependency. The knowledge that changes in heart rate lead to QT interval changes only after an appreciable time lag is only some decades-old (Franz et al., 1988; Lau et al., 1988). Although this dynamic aspect of the QT/heart rate relationship is now well-established and shown in repeated reports of solid data (Malik et al., 2008a; Jacquemet et al., 2014), some simplistic approaches to QT/heart-rate dependency still ignore the dynamic aspect, e.g., by proposing that QT interval duration measured on a beat-to-beat basis should be related solely to the duration of the preceding RR interval (Fossa, 2017), although this leads to substantial inaccuracies (example in Figure 1).

Despite these simplistic proposals, two components of the heart rate influence on QT duration are presently distinguished, and for the description of both, heart rate is usually expressed by corresponding durations of RR intervals. The *adaptation steepness* of the QT/heart rate relationship, i.e., how much QT interval changes when the underlying heart rate accelerates or decelerates, is usually described as QT/RR adaptation. This needs to be distinguished from the *speed* of QT/heart rate relationship, i.e., how quickly QT duration adapts to heart rate changes. The term *QT/RR hysteresis* is customarily (albeit perhaps somewhat inaccurately) used for this dynamic component (Malik, 2014; Zhang et al., 2014; Malik et al., 2016; Gravel et al., 2017, 2018). Previous studies suggested that both components are independent of each other. That is, a subject whose QT duration changes in response to heart rate changes more than in other subjects (i.e., who has a steeper QT/RR slope) may have the hysteresis speed both faster and slower than other subjects (Malik et al., 2008a). This makes the process of correcting the underlying heart rate for QT/RR hysteresis independent from the process of correcting the QT duration for the underlying heart rate. Indeed, different approaches to correcting the heart rate for hysteresis have previously been combined with different QT/heart rate corrections (Cassani González et al., 2012; Malik et al., 2016; Hnatkova et al., 2019a).

Although different studies have addressed the properties of QT/RR hysteresis, gaps still exist in the understanding of this phenomenon. Among others, the question remains open whether the delay needed to reach QT equilibrium after heart rate changes is driven by time or the number of cardiac cycles. In relation to this, it has not been fully established whether under physiologic conditions, the adaptation speed is the same when heart rate accelerates (and QT interval shortens) and when heart rate decelerates (and QT interval prolongs). Limited knowledge is also available on other physiologic characteristics of QT/RR hysteresis, including influence of sex and age.

Bearing these knowledge gaps in mind, we have investigated the properties of QT/RR hysteresis by QT interval and heart rate measurements in a large collection of long-term electrocardiographic (ECG) recordings collected in healthy subjects. The recordings were obtained in strictly physiologic conditions of controlled clinical studies and, thus, allowed to address the characteristics of the phenomenon without any pharmacologic or instrumental interventions.

## Methods

### Investigated Population and Electrocardiographic Recordings

Clinical pharmacology studies enrolled 751 healthy volunteers including 336 females, with no statistical age differences between females and males (34.5 ± 10.3 vs. 34.2 ± 8.7 years).

Before study enrolment, all the subjects had normal standard clinical ECG and normal clinical investigation. Standard inclusion and exclusion criteria mandated for phase I pharmacology studies (Guideline, 2001) were applied. These included negative tests for recreational substance tests and negative pregnancy tests in females. The studies were conducted at six different locations, and all were ethically approved by the institutional ethics bodies (Focus in Neuss; California Clinical Trials in Glendale; Parexel in Baltimore and Bloemfontein; PPD in Austin; and Spaulding in Milwaukee). The enrolled subjects gave informed written consent not only for study participation but also for scientific investigation of collected data. Parts of the same data have already been used in independent physiology studies assessing different electrocardiography aspects (Andršová et al., 2020; Toman et al., 2020; Hnatkova et al., 2021).

In every subject, repeated long-term 12-lead Holter ECG recordings with Mason-Likar electrode positions were obtained covering full day-time periods when the volunteers were on no medication and when they were not allowed to smoke and/or consume alcohol or caffeinated drinks. Because this investigation utilised only drug-free data either before the subjects received any investigative drug or after an appropriately long washout of the investigated drug and of its metabolites (Guideline, 2001), other specifics of the source pharmacology studies are unimportant.

### Electrocardiographic Measurement

Using previously described methods (Malik et al., 2008b, 2012), multiple 10-s sinus rhythm segments (i.e., segments without any ectopic beats) were extracted from the 12-lead Holter recordings. The segments captured different heart rates available in the Holter recordings including different heart rate changes.

In each of these ECG segments, QT interval was measured following published procedures (Malik et al., 2008b, 2012) that included repeated visual controls of all the measurements and assurance that corresponding ECG morphologies were interpreted in a consistent way (Hnatkova et al., 2009). The visually verified QT interval measurements were made in representative median waveforms of the 10-s segments (sampled at 1,000 Hz) with superimposition of all the 12 leads on the same isoelectric axis.

For each 10-s segment, the RR interval sequence within the segment and within the preceding 5 min of the Holter recordings was also obtained. The positions of QRS complexes were determined automatically using different previously validated detection algorithms. When these algorithms disagreed, the QRS were localised manually by simultaneous display of all the 12 ECG leads.

### QT/RR Adaptation Models

Since QT/heart rate relationship is known to be different in different individuals, the description of QT/RR adaptation used mathematical forms with parameters optimised for different subjects. All the following formal descriptions of the models expect the QT and RR interval durations to be expressed in seconds.

The principal analysis was based on the previously published (Malik et al., 2013) non-linear curvature regression model


$$\begin{array}{l} \text{Q}{\text{T}}_{\text{i}}=\text{X }+\text{Ψ}\left[{\left({\text{RR}}_{i}^{H}\right)}^{\gamma }-1\right]+\varepsilon_{i} \end{array}$$

where QT_i_ is the *i*-th measured QT interval; ${\text{RR}}_{i}^{H}$ is the corresponding hysteresis-corrected (see the next section) duration of the RR interval representing the underlying heart rate; X, Ψ, and γ are individually optimised parameters (γ is the curvature of the QT/RR relationship) and ε_*i*_ are zero-centred regression errors. As previously explained (Malik et al., 2013), the slope of regression is expressed as Ψ × γ (where X and Ψ are obtained by simple linear regression modelling for a given value of γ≠0, whilst the value of γ is obtained by minimising the mean of ε_*i*_ squares).

With this curvature model, individually corrected QTc intervals were given by the formula


$$\begin{array}{l} \text{QTc = QT+ }\Psi \left[\text{1- }{\left({\text{RR}}^{\text{H}}\right)}^{\gamma }\right] \end{array}$$

In addition to this primary curvature model of the QT/heart rate relationship, two simpler models were used to allow for comparisons with previous publications. The linear model was given by the formula


$$\begin{array}{l} \text{Q}{\text{T}}_{\text{i}}=\;\chi +\beta \left(RR_{i}^{H}-1\right)+\varepsilon_{i} \end{array}$$

while the log-log model was given by the formula


$$\begin{array}{l} \log({\text{QT}}_{i})=\;\phi +\;\alpha \times \log({\text{RR}}_{i}^{H})+\varepsilon_{i} \end{array}$$

where β, χ, α, and ϕ are individually optimised parameters and other symbols have the same meaning as in the curvature model. The parameters β and α are the slopes of the linear and log-log models, respectively.

The linear and log-log models led to QTc formulas:


$$\begin{array}{l} \text{QTc}=\text{QT}+\text{β}\left(1-\text{R}{\text{R}}^{\text{H}}\right)\text{ and QTc}=\text{QT}/{\left(\text{R}{\text{R}}^{\text{H}}\right)}^{\text{α}},\text{ respectively}. \end{array}$$

### Expressions of QT/RR Hysteresis

To distinguish QT/RR hysteresis driven by absolute time and by the number of preceding cardiac cycles, two models of QT/RR hysteresis were used. Both derived hysteresis-corrected RR^H^intervals as weighted averages of RR intervals preceding the QT measurement, and both used averaging weights based on previously proposed exponential decay, i.e., on the assumption that the influence of RR interval duration of the QT interval duration decreases exponentially when moving backward from the QT interval measurement (Malik et al., 2008a). That is, both models considered the history ${\left\{\text{R}{\text{R}}_{\text{i}}\right\}}_{\text{i}=0}^{\text{N}}$ of N consecutive RR intervals preceding the QT measurement (RR_0_ being closest to the QT measurement) and expressed the hysteresis-corrected RR^H^ interval as:


$$\begin{array}{l} {\text{RR}}^{H}=\overset{N}{\underset{i=0}{\sum }}\omega_{i}{\text{RR}}_{i}\text{ where}\overset{N}{\underset{i=0}{\sum }}\omega_{i}=\;1. \end{array}$$

The models differed in the definition of weights ${\left\{\omega_{i}\right\}}_{i=0}^{N}$. The model assuming the dependency on the number of preceding RR intervals used weights such that


$$\begin{array}{l} \overset{k}{\underset{i=0}{\sum }}\omega_{i}=\frac{1-e^{{𝔏}_{I}\left(\frac{k+1}{N+1}\right)}}{1-e^{{𝔏}_{I}}}\text{ for each }k,0\leq k\leq N, \end{array}$$

while the model assuming the dependency on the absolute time preceding the QT interval measurement used the weights such that


$$\begin{array}{l} \;\overset{k}{\underset{i=0}{\sum }}\omega_{i}=\frac{1-e^{{𝔏}_{T}\left(\frac{T\left(k\right)}{T\left(N\right)}\right)}}{1-e^{{𝔏}_{T}}}\text{where}\\ T\left(k\right)=\;\overset{k}{\underset{i=0}{\sum }}RR_{i}\text{ for each }k,0\leq k\leq N. \end{array}$$

The coefficients 𝔏_*I*_ and 𝔏_*T*_were individually optimised.

After the QT/RR hysteresis models were individually optimised (see the subsequent section), the coefficients 𝔏_*I*_ and 𝔏_*T*_ were, for the purposes of physiologic interpretation, converted to 95% hysteresis constants ℭ_*I*_ and ℭ_*T*_, i.e., for each subject, the constant ℭ_*I*_ specified the number of RR intervals after which the dynamicity of QT interval duration reached 95% of the rate-corresponding change, and likewise, the constant ℭ_*T*_ specified the time needed for the 95% dynamic QT interval change. That is, the interval count ℭ_*I*_ and time span ℭ_*T*_ were identified such that:


$$\begin{array}{l} \frac{1-e^{{𝔏}_{I}\left(\frac{{ℭ}_{I}+1}{N+1}\right)}}{1-e^{{𝔏}_{I}}}=0.95\text{ and }\frac{1-e^{{𝔏}_{T}\left(\frac{{ℭ}_{T}}{T\left(N\right)}\right)}}{1-e^{{𝔏}_{T}}}=\;0.95. \end{array}$$

This means that the lower the count ℭ_*I*_ or the shorter the time span ℭ_*T*_, the faster the QT-interval adaptation to heart rate changes (or, in other words, the shorter the delay after which the QT interval reaches equilibrium after heart rate change).

### Residuals of QT/RR Models and Individual Model Optimisation

The combination of the three QT/RR adaptation models with the two QT/RR hysteresis models led to six different possibilities. In each subject, each of the six possibilities was optimised to achieve the closes fit of the QT/RR^H^ regressions. That is, for each subject, parameters, Ψ, γ, β, α, 𝔏_*I*_, and 𝔏_*T*_ were optimised such that each of the models led to the lowest standard deviation of the corresponding QTc intervals. These values were expressed in milliseconds.

By definition of the *QTc* intervals, the QTc standard deviations constituted regression residuals of the intra-subject QT/RR^H^ regression models, which were used to compare the closeness of fit among the six different modelling possibilities. This was based on the understanding that the closeness of data fit is an appropriate measure of the relevancy of any physiologic model. In other words, a combination of QT/RR adaptation and hysteresis models was considered physiologically more valid than another combination if it led to significantly lower regression residuals. Since the regression residuals were calculated for each subject separately, their intra-subject means were compared across the study population.

### Distinction Between Heart Rate Acceleration and Deceleration

To investigate whether QT/RR hysteresis speed differs between heart rate acceleration and deceleration, episodes of systematic rate increase and decrease were identified in the recordings of each study subject. For this purpose, ${\left\{\text{R}{\text{R}}_{\text{i}}\right\}}_{\text{i}=0}^{\text{N}}$ histories of RR intervals preceding each QT measurement were considered and the slopes 𝔰_0−30_, 𝔰_30−60_, and 𝔰_0−60_of a linear regression between *RR*_*i*_ durations and their index numbers *i* were calculated between 0 and 30 s, 30 and 60 s, and 0 and 60 s preceding the QT measurement, respectively.

QT interval measurement was considered preceded by heart rate acceleration if 𝔰_0−30_>0, 𝔰_30−60_>0, and if the lower 95% confidence intervals of 𝔰_0−60_ was positive. Likewise, QT interval measurement was considered preceded by heart rate deceleration if 𝔰_0−30_ <0, 𝔰_30−60_ <0, and if the upper 95% confidence intervals of 𝔰_0−60_ was negative (note that *RR*_0_ interval was closest to the QT measurement, and that while 5-min histories of RR interval were available, the rate acceleration and deceleration episodes were defined using only the preceding 1 min. This is consistent with the asymptotic nature of the ω_*i*_ coefficients in the exponential decay models).

In each subject, QT measurements preceded by heart rate acceleration and deceleration according to this definition were sorted according to the corresponding 𝔰_0−60_ slopes. A stepwise elimination algorithm was used to obtain subsets of these QT interval measurements that were as close as possible to a one-to-one correspondence between the absolute |𝔰_0−60_| values. This eliminated the possibility of comparing QT/RR hysteresis speeds among data that showed faster rate acceleration than deceleration or vice versa.

In each of these balanced sets of heart rate acceleration and deceleration episodes, the optimisation of QT/RR hysteresis models, as described previously, was repeated (that is, separately for acceleration and deceleration episodes), and for each subject and for each of the six combinations of QT/RR adaptation and hysteresis models, separate rate acceleration and deceleration coefficients 𝔏_*I*_ and 𝔏_*T*_ were obtained and converted into acceleration and deceleration hysteresis constants ℭ_*I*_ and ℭ_*T*_. Their statistical evaluations allowed for the comparison of QT/RR hysteresis speed between rate increase and decrease.

### Statistics and Data Presentation

Descriptive data are presented as means ± SD. Comparisons between females and males were based on two-sample two-tail *t*-test assuming different variations between compared datasets, intra-subject comparisons (e.g., comparisons of regression residuals among the different QT/RR adaptation models) were based on paired two-tail *t*-test. The significance of linear regression slopes between age and investigated indices was tested by Fisher–Snedecor F distribution. Statistical tests used IBM SPSS package version 27. *P*-values below 0.05 were considered statistically significant. Because of interdependence among the different indices, no correction for multiplicity of statistical testing was performed.

## Results

**Figure 2:**
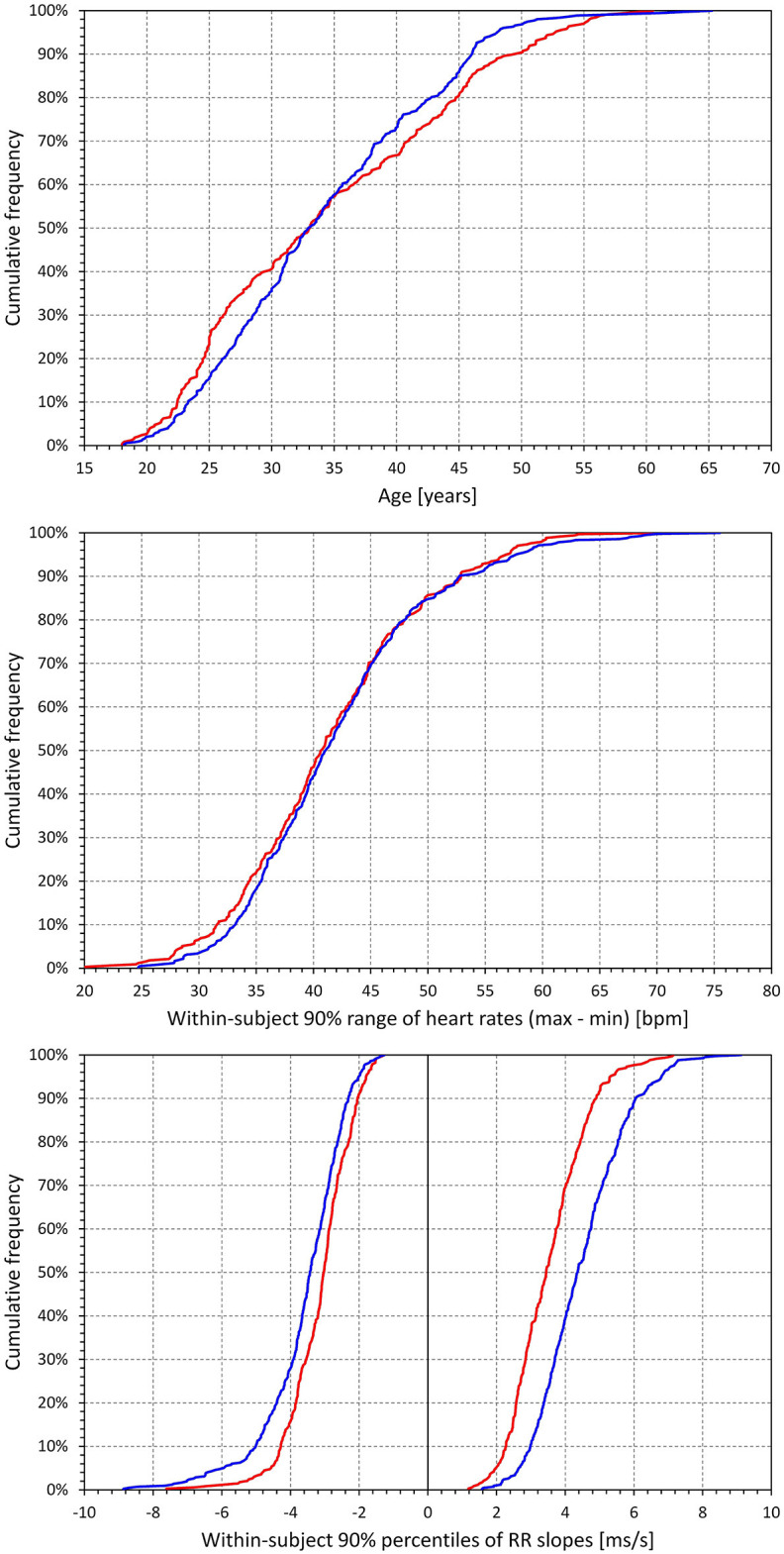
Characteristic of the study population and electrocardiographic measurements. Top panel shows cumulative distributions of the ages of investigated subjects. The middle panel shows cumulative distributions of the 90% ranges of intra-subject heart rates in study subjects (that is, in each study subject, difference between the 5th and 95th percentiles of heart rates at which the QT interval was measured was calculated, and the panel shows the cumulative distribution of these differences). The bottom panel shows the cumulative distributions of the intra-subject 5th and 95th percentiles of the RR interval slopes preceding QT interval measurements (that is, for each subject, 60-s slopes of RR interval durations vs. their index numbers were obtained for each RR history of QT interval measurements, and the graphs show the distribution of the intra-subject 5th and 95th percentiles of these slopes. In each subject, the 5th percentile corresponded to heart rate deceleration, distributions below 0, and the 95th percentile corresponded to heart rate acceleration, distributions above 0). In each graph, the red and blue lines correspond to the female and male subjects, respectively.

Relevant characteristics of the study population are summarised in Figure 2. As seen in this Figure, most of the subjects were aged between 20 and 50 years (87.8% females and 94.6% males).

In these subjects, altogether, 897,570 QT interval measurements in separate 10-s ECG segments were made, each with a 5-min history of preceding RR intervals. On average, there were 1,195 ± 293 such measurements in the individual subjects. Figure 2 also shows that in the individual subjects, broad ranges of heart rates were covered by these measurements, assuring that the data were suitable for stable regression analyses between QT interval and the underlying RR interval expressions (indeed, all the QT/RR models reported further led to significantly positive QT/RR slopes). Finally, Figure 2 shows that in the individual subjects, the 5-min histories of preceding RR intervals covered sufficient ranges of heart rate acceleration and deceleration suitable for the QT/RR hysteresis estimates. On average, 74.3 ± 7% of QT interval measurements in females and 72.4 ± 7.4% of QT interval measurements in males were preceded by RR interval histories that showed statistically significant acceleration or deceleration (i.e., within 1 min preceding the QT interval measurement, the slope between RR interval durations and their order numbers was significantly different from zero).

### QT/RR Adaptation Models

**Figure 3:**
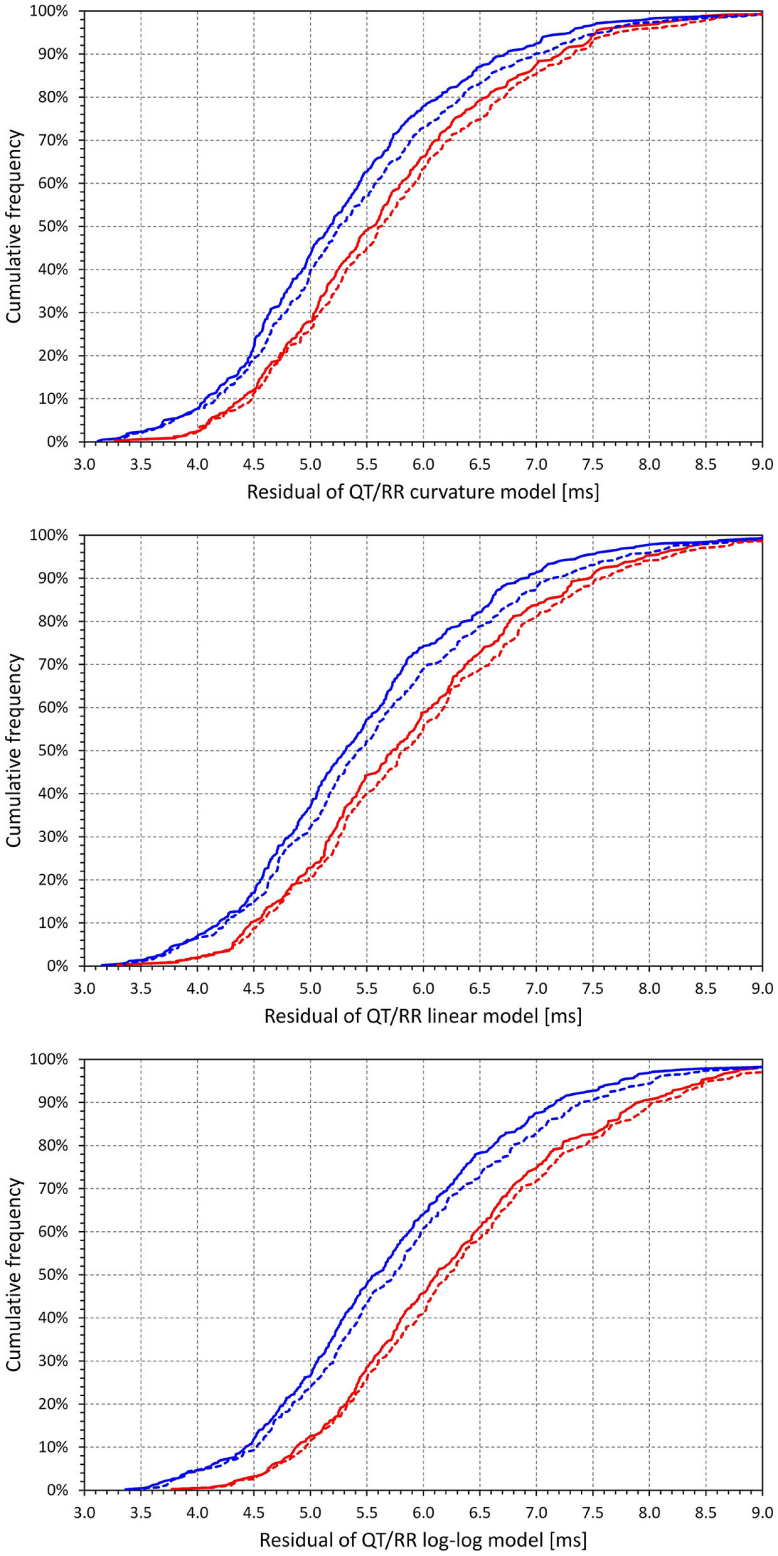
Cumulative distributions of intra-subject residuals of QT/RR^H^ regression models for the curvature model (top panel), linear model (middle panel), and log-log model (bottom panel). In each of the panels, the red and blue lines correspond to females and males, respectively; the full and dashed lines correspond to the time-based and interval-based QT/RR hysteresis models, respectively.

Figure 3 shows cumulative distributions of regression residual values for all the three types of QT/RR adaptation models. It is clearly visible that for all the three models, the regression residuals were larger in females than in males. For the combination of curvature QT/RR adaptation with time-based and interval-based QT/RR hystereses, the residuals in females and males were 5.68 ± 1.1 vs. 5.31 ± 1.08 ms, and 5.77 ± 1.13 vs. 5.4 5± 1.14 ms, respectively, and both *p*-values were <0.0001. The same strong statistical significance (*p* < 0.0001) of sex differences was seen for all the other model combinations.

Comparison of the different panels in Figure 3 also shows that, not surprisingly, regression residuals of the curvature QT/RR model were smaller than those of the linear QT/RR model, and that the residuals of the log-log QT/RR model were noticeably larger than those of the other models. In combination with the time-based QT/RR hysteresis, the residuals over the complete population were 5.47 ± 1.1, 5.65 ± 1.14, and 5.98 ± 1.21 ms for the curvature, linear, and log-log models, respectively. In combination with the interval-based QT/RR hysteresis, the corresponding values were 5.59 ± 1.15, 5.78 ± 1.19, and 6.1 ± 1.25 ms.

**Figure 4:**
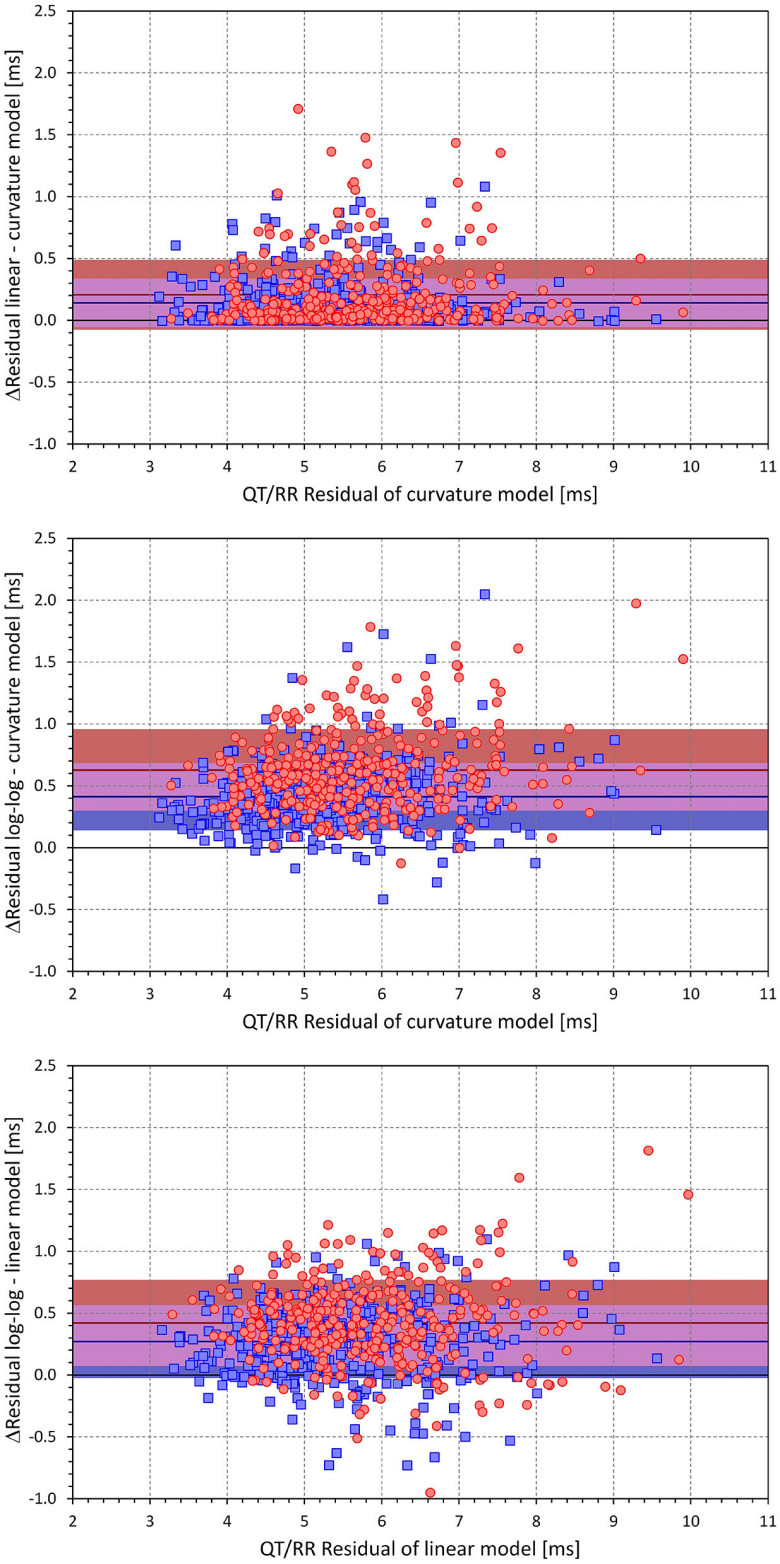
Scatter diagrams of the intra-individual differences among the different QT/RR^H^ regression models (all combined with the time-based QT/RR hysteresis model). The top panel shows the differences between the residuals of the linear and curvature models and the residuals of the curvature model, the middle panel shows the differences between the residuals of the log-log and curvature models and the residuals of the curvature model, and the bottom panel shows the differences between the residuals of the log-log and linear models and the residuals of the linear model. In each panel, the red circle and blue square marks correspond to females and males, respectively. The red and blue horizontal lines show the mean values of the residual differences in females and males, respectively. The light-coloured red and blue bands show the spans of mean ± standard deviation of the residual differences in females and males, respectively; the light-coloured violet bands show the overlap of the mean ± standard deviation bands between both sexes.

Figure 4 shows scatter diagrams of intra-subject differences between the regression residuals of the different QT/RR adaptation models combined with the time-based QT/RR hysteresis model. The population averages of the intra-subject differences among the curvature and linear, curvature and log-log, and linear and log-log models were 0.171 ± 0.240, 0.509 ± 0.317, and 0.338 ± 0.328 ms, respectively (all significantly positive with *p* < 0.0001). Similar statistical differences were also seen in each model separately for females and males, and for the combination with the interval-based QR/RR hysteresis model.

**Figure 5:**
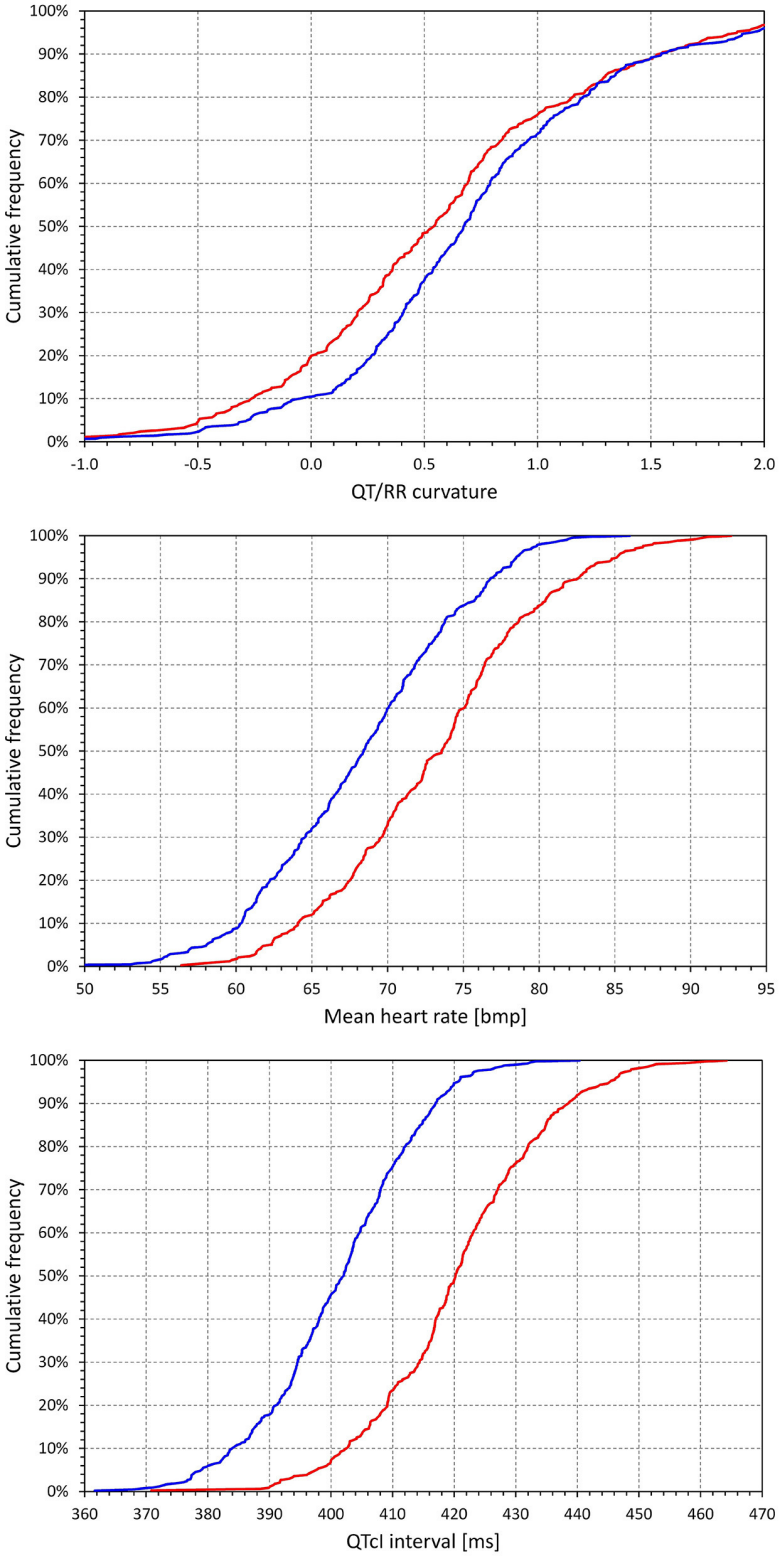
Cumulative distributions of the subject-specific characteristics of the curvature QT/RR^H^ model combined with time-based curvature QT/RR hysteresis model. The distributions of model curvature (parameter **γ**), mean heart rate derived from the RR^H^ values, and mean individually corrected QTc intervals are shown in the top, middle, and bottom panels, respectively. The red and blue lines correspond to females and males, respectively.

Using the curvature QT/RR adaptation model combined with the time-based QT/RR hysteresis model, Figure 5 shows differences between females and males for the curvature parameter of the adaptation model (0.579 ± 0.709 vs. 0.742 ± 0.733, *p* = 0.0021), heart rate corresponding to the intra-subject mean *RR*^*H*^ intervals (73.3 ± 6.8 vs. 68.3 ± 6.4 beats per minute, *p* < 0.0001), and the intra-subject mean of QTc intervals (421 ± 14 vs. 401 ± 13 ms, *p* < 0.0001).

**Figure 6:**
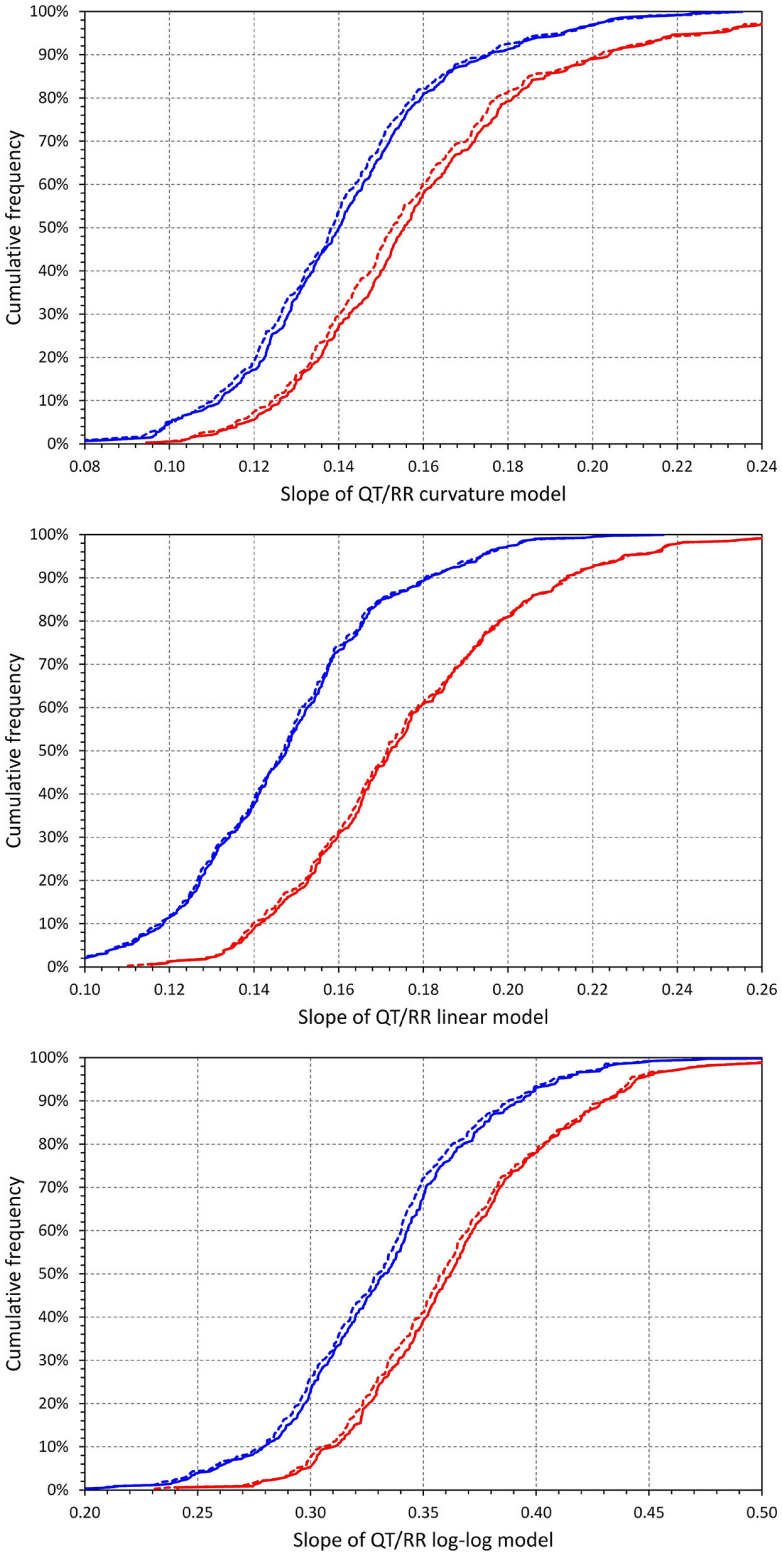
Cumulative distributions of intra-subject slopes of QT/RR^H^ regression models for the curvature model (top panel), linear model (middle panel), and log-log model (bottom panel). In each of the panels, the red and blue lines correspond to females and males, respectively; the full and dashed lines correspond to the time-based and interval-based QT/RR hysteresis models, respectively.

Figure 6 confirms previously published observations that irrespective of adaptation and hysteresis models, the pattern of QT/RR relationship is steeper in females than in males (Malik et al., 2013) (for example, the slope of the curvature adaptation model combined with time-based hysteresis was 0.161 ± 0.033 in females and 0.142 ± 0.026 in males, *p* < 0.0001).

### QT/RR Hysteresis Models

**Figure 7:**
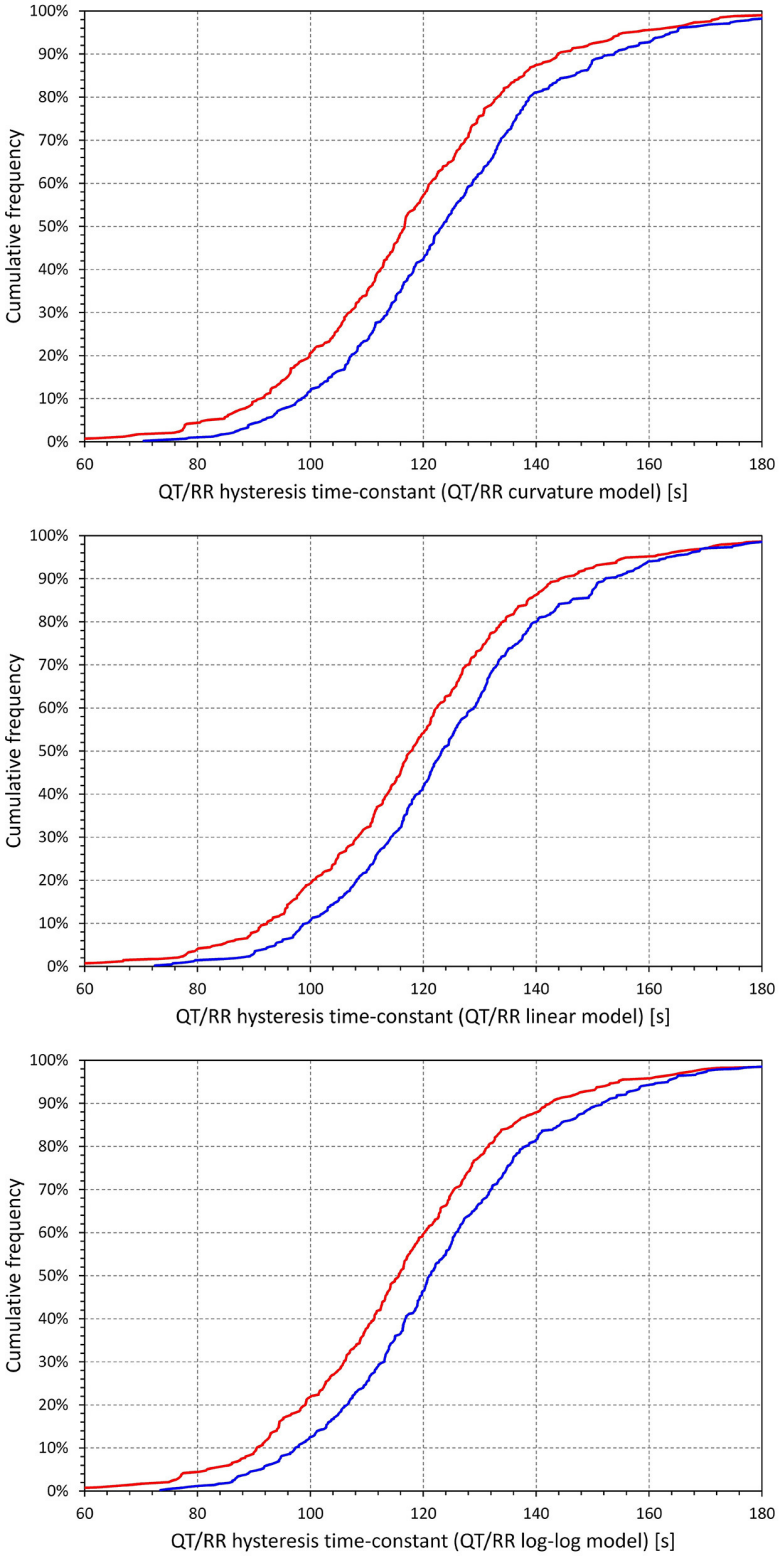
Cumulative distributions of QT/RR hysteresis time-constants derived from the combination with the QT/RR^H^ curvature model (top panel), linear model (middle panel), and log-log model (bottom panel). In each of the panels, the red and blue lines correspond to females and males, respectively.

**Figure 8:**
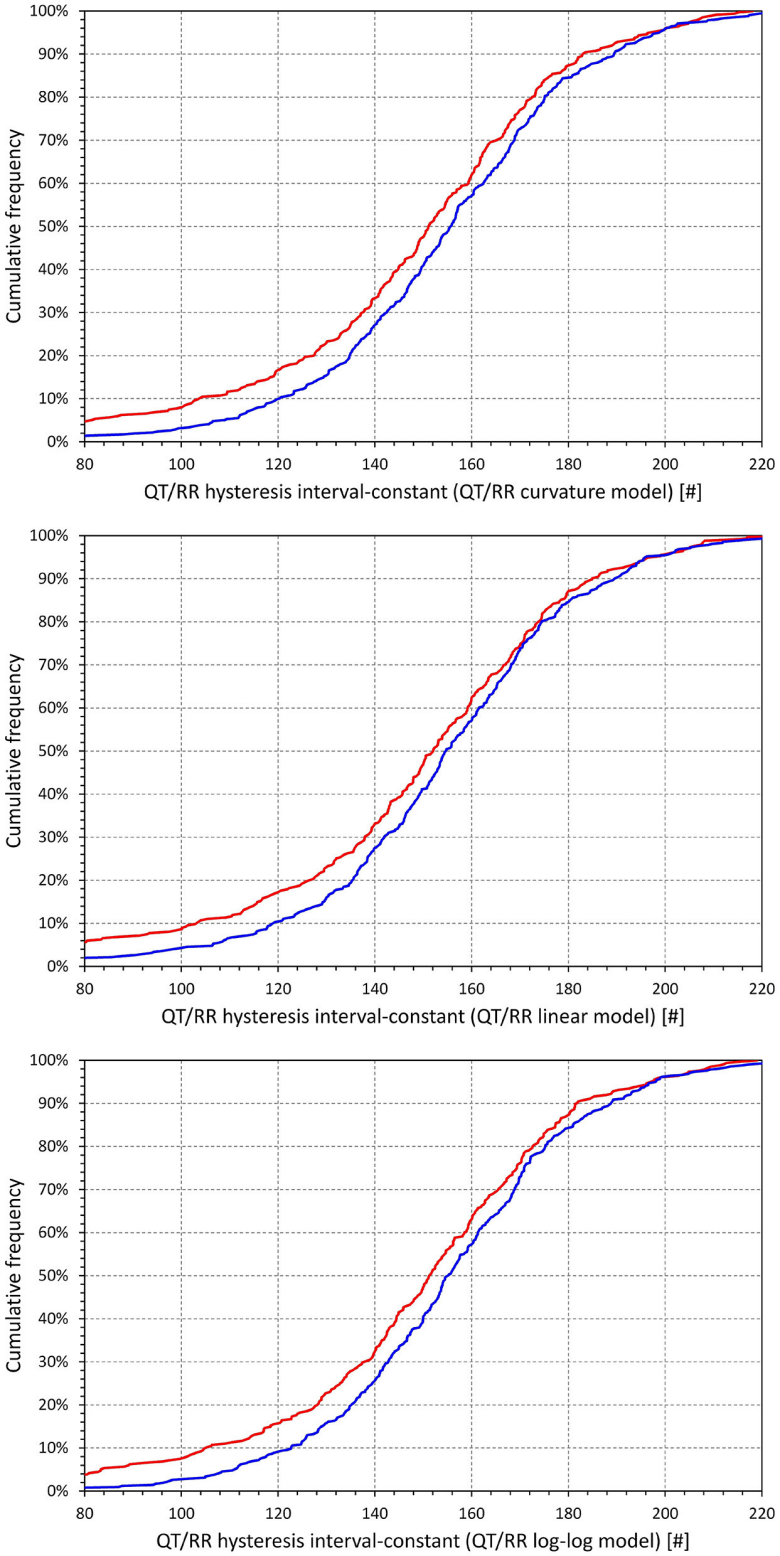
Cumulative distributions of QT/RR hysteresis interval constants derived from the combination with the QT/RR^H^ curvature model (top panel), linear model (middle panel), and log-log model (bottom panel). In each of the panels, the red and blue lines correspond to females and males, respectively.

Figure 7 shows that irrespective of the QT/RR adaptation model, the QT/RR hysteresis time-constants ℭ_*T*_ were, on average, shorter in females than in males (e.g., 118 ± 22 vs. 125 ± 22 s and *p* < 0.0001 for the QT/RR curvature model). Figure 8 shows that the same sex difference was also present for the hysteresis interval-constants ℭ_*I*_ (e.g., 148 ± 32 vs. 155 ± 27, *p* = 0.0027, also for the QT/RR curvature model). The somewhat lesser sex difference in the ℭ_*I*_ constants compared to ℭ_*T*_ constants needs to be interpreted together with the faster rates in females (as shown in Figure 4), which led to the same number of RR intervals representing shorter time in females than in males.

**Figure 9:**
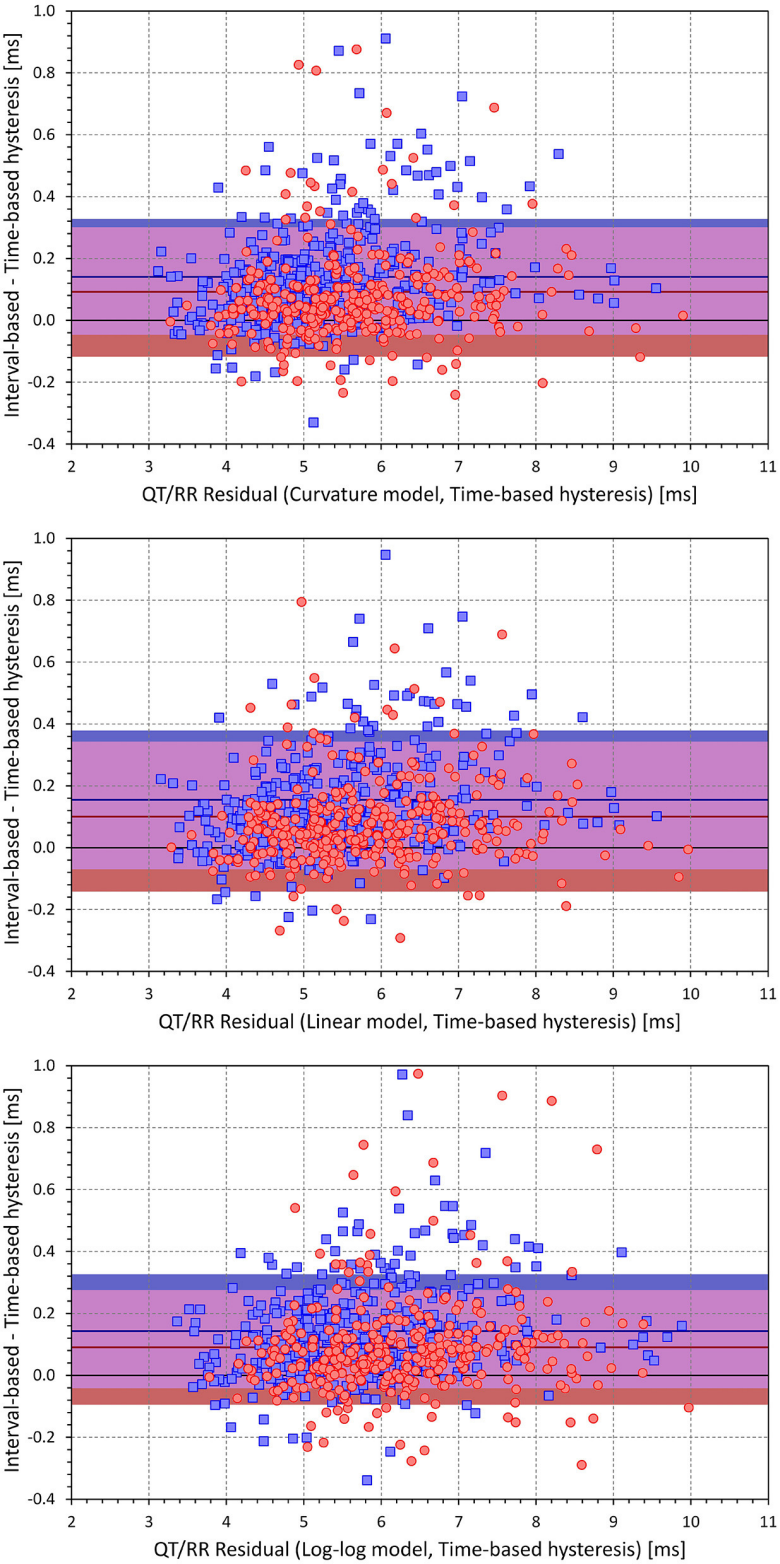
Scatter diagrams of the intra-individual differences among regression residuals comparing the interval-based and time-different QT/RR hysteresis models. The differences between the residuals and the residuals of the time-based QT/RR hysteresis model are shown for the QT/RR^H^ curvature model in the top panel, linear model in the middle panel, and log-log model in the bottom panel. In each panel, the red circle and blue square marks correspond to females and males, respectively. The red and blue horizontal lines show the mean values of the residual differences in females and males, respectively. The light-coloured red and blue bands show the spans of mean ± standard deviation of the residual differences in females and males, respectively; the light-coloured violet bands show the overlap of the mean ± standard deviation bands between both sexes.

As already seen in Figure 3, a combination of any of the QT/RR adaptation models with the time-based QT/RR hysteresis led to lower regression residuals compared to the combination with the interval-based hysteresis. Confirmation of this observation with intra-subject differences is shown in Figure 9. The population averages of intra-subject differences in the residuals were 0.119 ± 0.198, 0.131 ± 0.234, and 0.119 ± 0.186 ms for the curvature, linear, and log-log adaptation models, respectively, and all were significantly positive (*p* < 0.0001). In all the three adaptation models, these intra-subject differences were also smaller in females compared to males (e.g., 0.092 ± 0.209 vs. 0.141 ± 0.187ms and *p* = 0.001 with the curvature adaptation model).

### Relationship to Age

**Figure 10:**
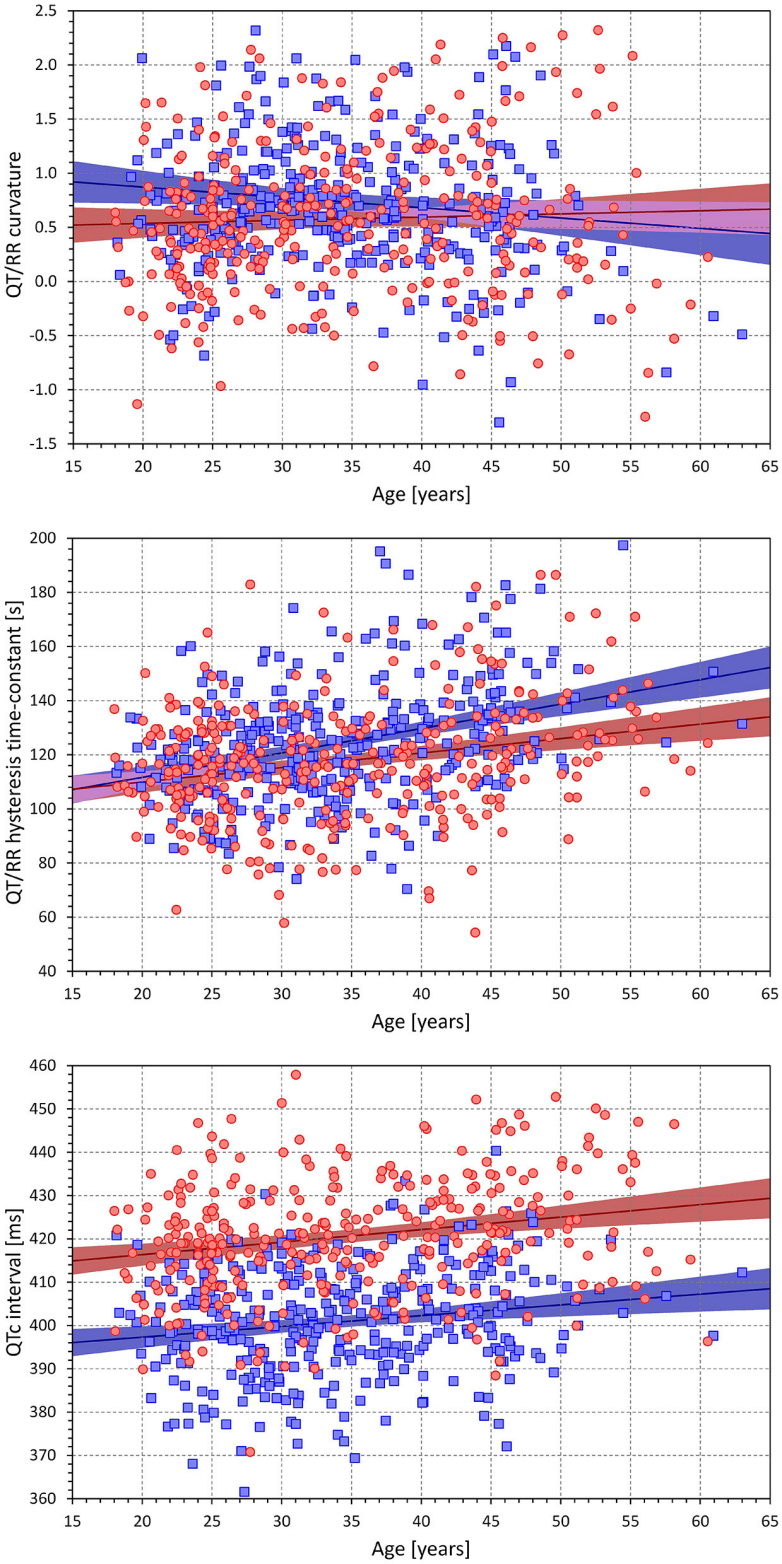
Scatter diagrams showing age influence on the subject-specific curvatures of the QT/RR^H^ curvature model combined with time-based QT/RR hysteresis (top panel), on the subject-specific hysteresis time-constants of the QT/RR^H^ curvature model (middle panel), and on the mean of individually corrected QTC intervals of the QT/RR^H^ curvature model combined with time-based QT/RR hysteresis (bottom panel). In each panel, the red circle and blue square marks correspond to females and males, respectively. The red and blue lines show the linear regression models between the displayed characteristics and age, the light-coloured red and blue bands show the 95% confidence intervals of the linear regressions, and the light-coloured violet areas show the overlaps among the regression confidence intervals of both sexes.

Figure 10 shows the relationship to age of selected indices derived from the curvature QT/RR adaptation model combined with the time-based QT/RR hysteresis model. The Figure shows that the curvature model was negatively moderately related to age in the males (*p* = 0.039) but not in the females, and that hysteresis time constant was significantly increased with age (*p* < 0.0001 for both sexes), as did the mean QTc interval (*p* < 0.0001 in females and *p* = 0.001 in males). Whilst there was little sex difference in QTc prolongation with increasing age, hysteresis time constant increased with age more steeply in males than in females (*p* = 0.03). That is, the sex difference in the hysteresis time constant increased with age. No other index showed a relationship to age (including, perhaps surprisingly, the regression residuals).

The same observation of hysteresis constants and individually corrected QTc intervals increasing with age was made with other combinations of the QT/RR adaptation and QT/RR hysteresis models.

### QT/RR Hysteresis During Rate Acceleration and Deceleration

In individual study subjects, the close one-to-one matching between heart rate acceleration and deceleration instances selected 66 ± 20 pairs of ECG segments with QT interval measurements.

**Figure 11:**
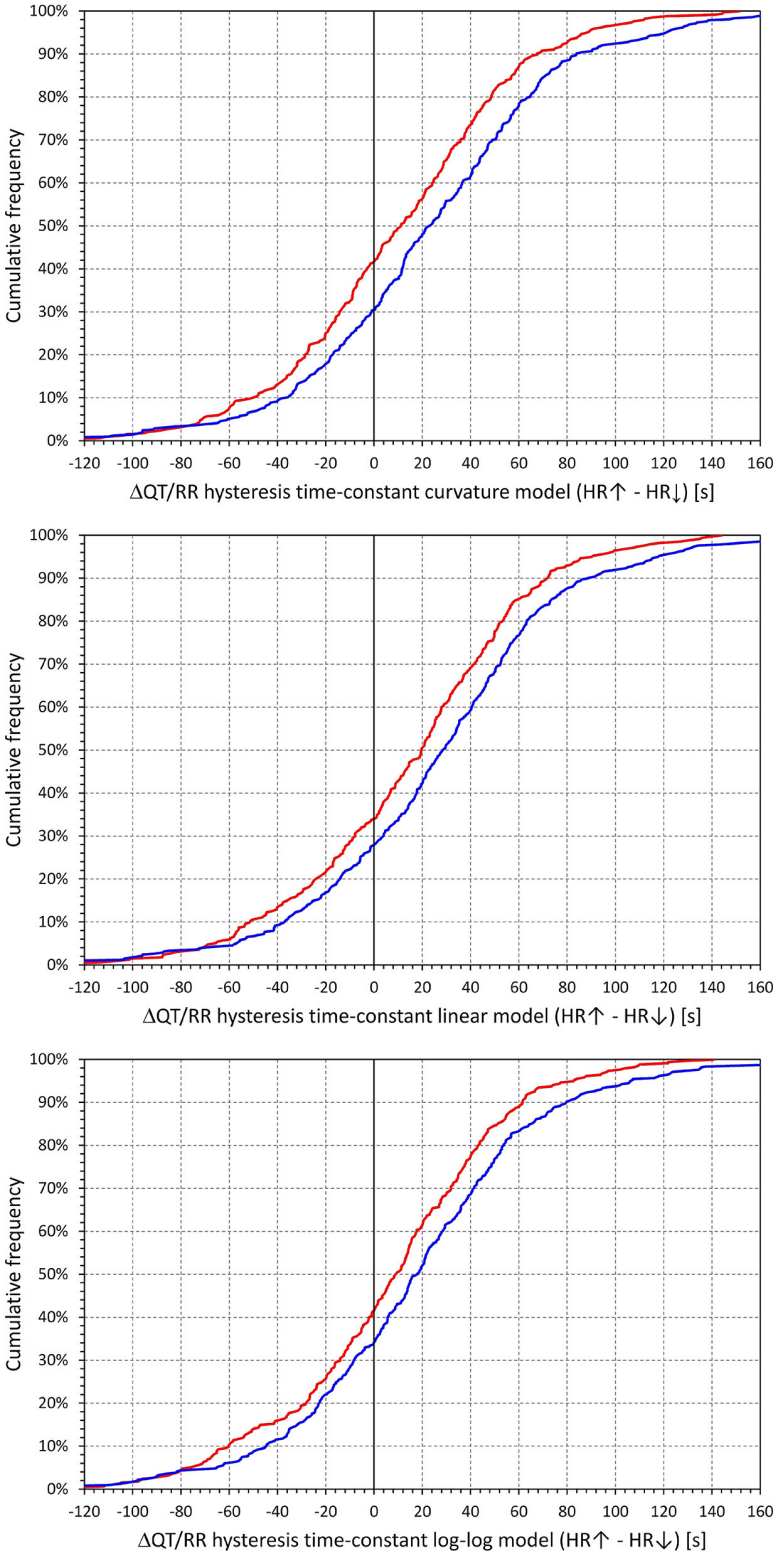
Cumulative densities of subject-specific differences among hysteresis time constants during heart rate acceleration and deceleration. The top, middle, and bottom panels show the differences for the curvature, linear, and log-log QT/RRH models, respectively. In each panel, the red and blue lines correspond to females and males, respectively.

**Figure 12:**
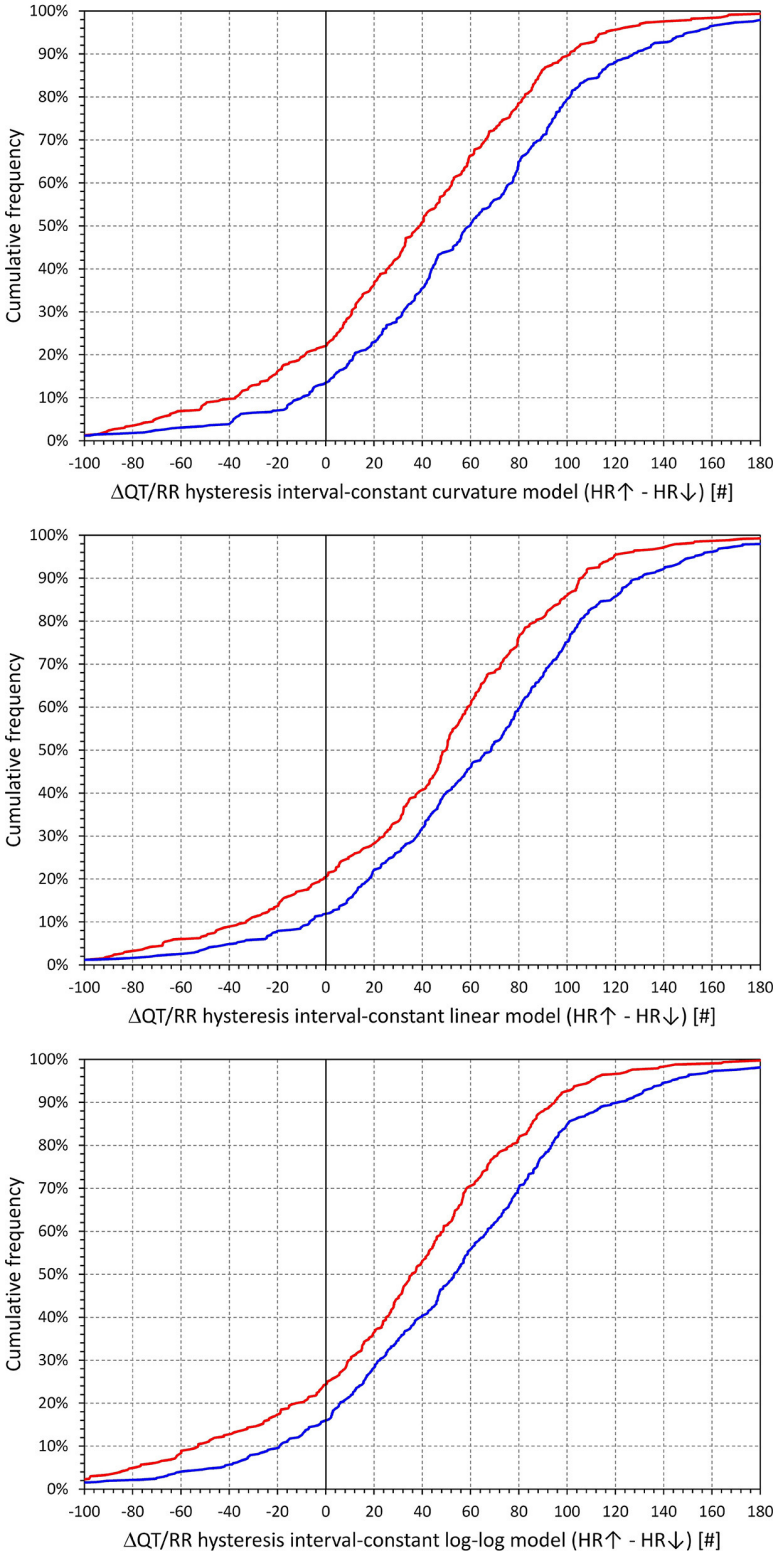
Cumulative densities of subject-specific differences among hysteresis interval constants during heart rate acceleration and deceleration. The top, middle, and bottom panels show the differences for the curvature, linear, and log-log QT/RR^H^ models, respectively. In each panel, the red and blue lines correspond to females and males, respectively.

The cumulative distributions of the differences between hysteresis time-constants ℭ_*T*_ optimised for rate acceleration and deceleration are shown in Figure 11; the same differences for the interval-constants ℭ_*I*_ are shown in Figure 12. Both Figures show that the subjects in whom the hysteresis speed was slower during heart rate acceleration than during heart rate acceleration were more numerous compared to the subjects showing the opposite relationship. The Figures also show that the difference in the proportion of subjects was more pronounced in males than in females.

**Figure 13:**
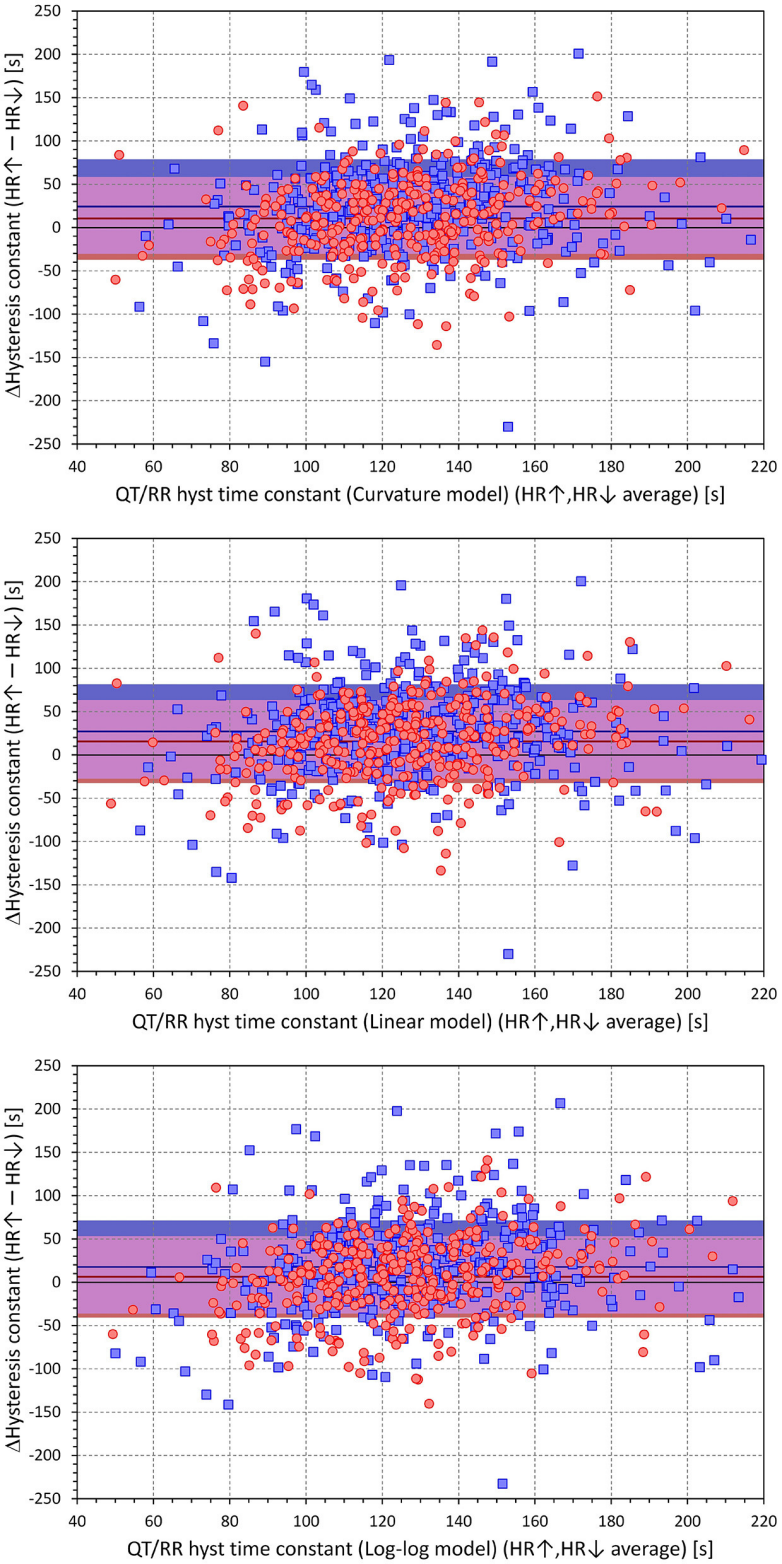
Scatter diagrams of subject-specific differences among hysteresis time constants during heart rate acceleration and deceleration plotted against their intra-subject averages. The top, middle, and bottom panels show the differences for the curvature, linear, and log-log QT/RR^H^ models, respectively. In each panel, the red circle and blue square marks correspond to females and males, respectively. The red and blue horizontal lines show the mean values of the residual differences in females and males, respectively. The light-coloured red and blue bands show the spans of mean ± standard deviation of the residual differences in females and males, respectively; the light-coloured violet bands show the overlap of the mean ± standard deviation bands between both sexes.

**Figure 14:**
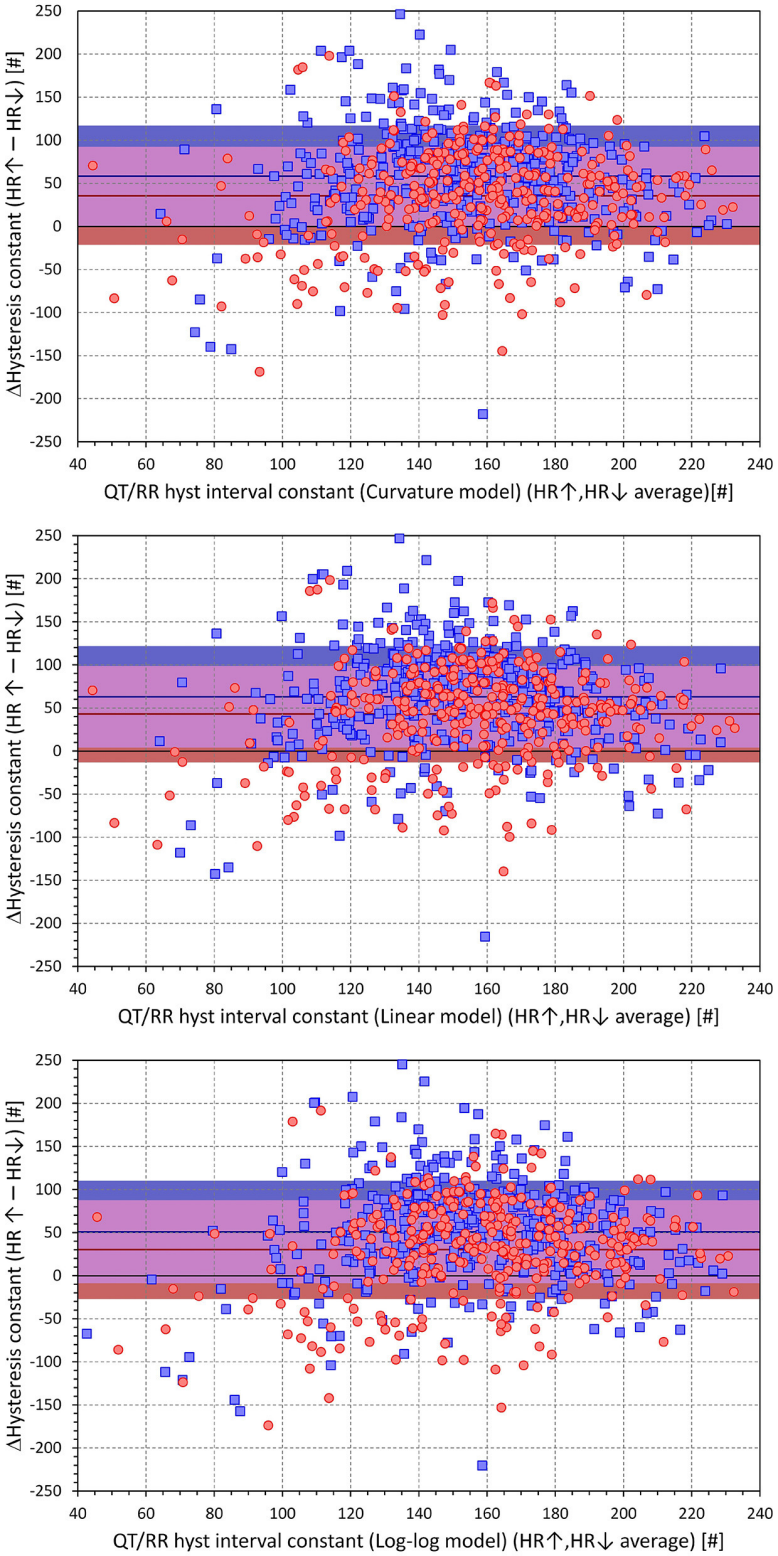
Scatter diagrams of subject-specific differences among hysteresis interval constants during heart rate acceleration and deceleration plotted against their intra-subject averages. The top, middle, and bottom panels show the differences for the curvature, linear, and log-log QT/RR^H^ models, respectively. In each panel, the red circle and blue square marks correspond to females and males, respectively. The red and blue horizontal lines show the mean values of the residual differences in females and males, respectively. The light-coloured red and blue bands show the spans of mean ± standard deviation of the residual differences in females and males, respectively; the light-coloured violet bands show the overlap of the mean ± standard deviation bands between both sexes.

The corresponding differences between hysteresis time and interval constants are shown in Figures 13, 14 that present the constants optimised for rate acceleration and deceleration in a Bland-Altman type of scatter diagrams. The population means (as well as sex-specific population means) of all the differences were significantly positive, although the diagrams also show outliers of the overall data distribution (mainly seen in cases with only few acceleration-deceleration data pairs). When optimising the QT/RR hysteresis models with the curvature models of QT/RR dependency, the acceleration-deceleration differences in the ℭ_*T*_ constants were 35.5 ± 56.8 and 58.4 ± 58.8 s in females and males, respectively, both significantly positive (*p* < 0.0001) and significantly different between sexes (*p* < 0.0001). The corresponding differences in the ℭ_*I*_ constants were 15.6 ± 47.8 and 27.2 ± 54.5 cardiac cycles, again both significantly positive (*p* < 0.0001) and significantly different between sexes (*p* = 0.0019).

## Discussion

The results of these analyses offer several conclusions of physiologic and, possibly, practical relevance. First, the profile of QT/RR hysteresis appears to be driven by absolute time rather than by the numbers of cardiac cycles. Second, the speed of QT/RR hysteresis is decreased with increase in age, and the duration of carefully and individually rate-corrected QTc interval is increased with increase in age. Third, QT/RR hysteresis differences between heart rate acceleration and deceleration are not physiologically systematic (i.e., they differ among different healthy subjects), but our data suggest that on average, QT/RR hysteresis speed is slower after heart rate acceleration than after heart rate deceleration. All these observations were based on the evaluation and comparisons of different combinations of the QT/RR adaptation and QT/RR hysteresis models.

### QT/RR Hysteresis Properties

**Figure 15:**
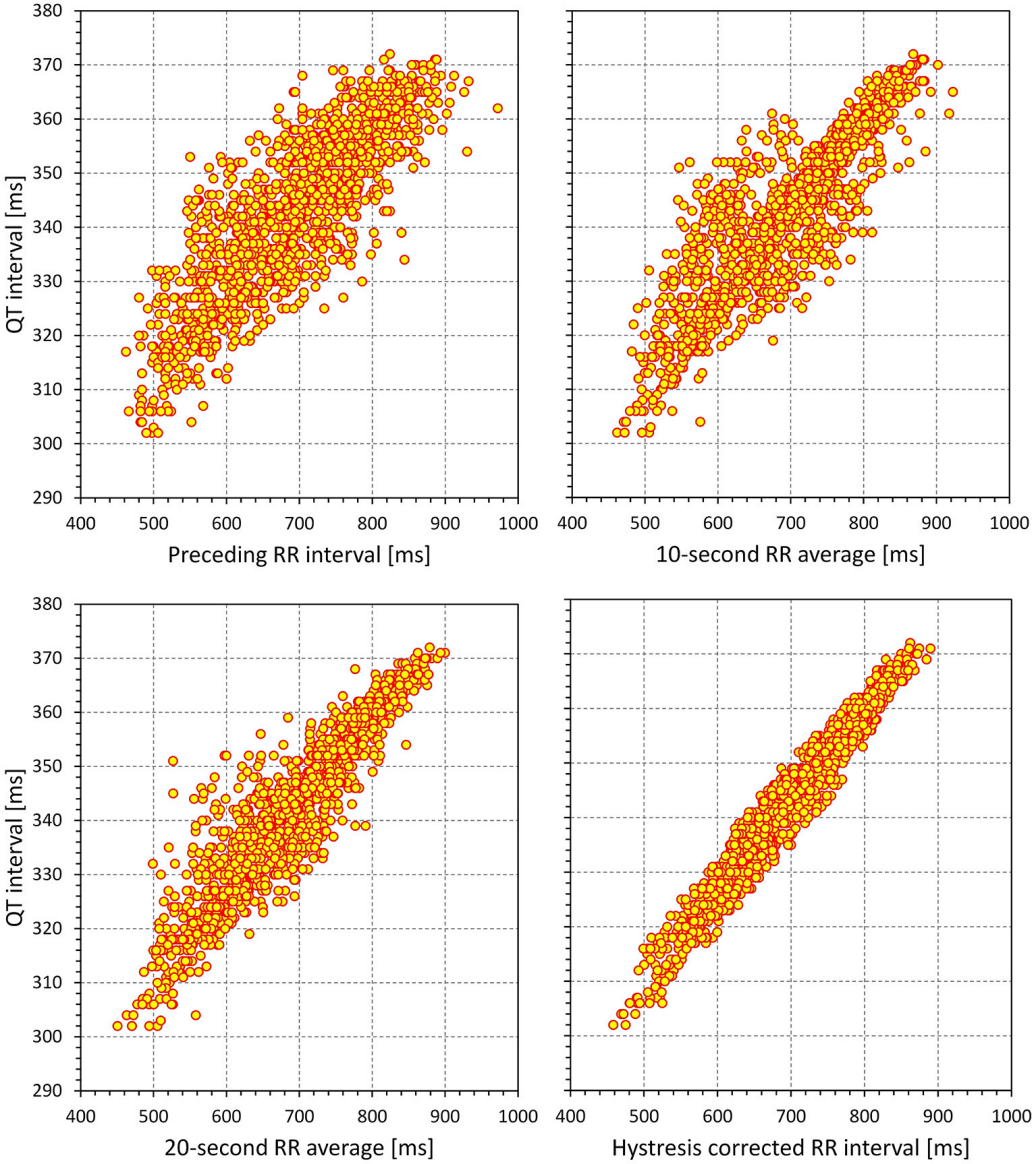
Example of the effect of incorporating QT/RR hysteresis correction to the study of QT/RR relationship. The data (1,440 separate measurements) were obtained in a 32-year-old female. The QT intervals (the same values in all panels) are related to the duration of RR interval preceding the QT measurement (top left panel), RR interval average over 10 s preceding the QT measurement (top right panel), RR interval average over 20 s preceding the QT measurement (bottom left panel), and RR interval duration corrected by a subject-specific exponential decay model of QT/RR hysteresis (bottom right panel). Note the changes in the compactness of the QT/RR relationship.

Repeated studies have previously described the implications of QT/RR hysteresis for the accuracy of the heart rate correction of QT interval measurement (Malik, 2014; Gravel et al., 2017, 2018). It has also been shown that incorporating hysteresis correction reduces the variability of QTc values significantly (Malik et al., 2008a,c). All the hysteresis estimates postulate that the RR interval duration used in the heart rate correction formulas of the QT interval cannot be directly measured but needs to be derived from a longer history of cardiac cycles preceding the QT measurement. This increases the compactness of the QT/RR relationship and allows for characterisation of the relationship in each subject with a greater precision and increased confidence (example in Figure 15).

Whilst the exponential decay model used in this study allowed us to distinguish the time-based and interval-based hysteresis expressions, other approaches have been proposed (Jacquemet et al., 2014), e.g., the autoregressive filter approach which, for beat-to-beat consecutive measurements of RR intervals ${\left\{RR_{i}\right\}}_{i=0}^{N}$, optimises a parameter ϑ so that QT interval measurement following the interval *RR*_*j*_ (for *j*≫0) is corrected for $RR_{j}^{H}=\vartheta RR_{j}+\left(1-\vartheta \right)RR_{j-1}^{H}$. It is easy to demonstrate that this autoregressive approach differs minimally from the interval-based exponential decay model (Malik, 2014). Making the parameter ϑ cycle-by-cycle dependent on the duration of RR intervals would lead to an approach closely corresponding to the time-based approach that our study found significantly (albeit numerically only slightly) more accurate. Therefore, implementation of our observation of the preference of the time-based QT/RR hysteresis models needs to be further tested in other mathematical implementations.

In addition to separate estimates of QT/RR hysteresis, i.e., corrections of the RR interval values to be used in QT/heart-rate corrections, more complex autoregressive models involve both beat-to-beat RR interval history and QT interval history (Porta et al., 2010, 2020; El-Hamad et al., 2019). These approaches were shown useful in providing estimates of QT interval changes and variability that cannot be explained by heart rate and cardiac period influence (Baumert et al., 2016). We were not able to employ any of these methods, since QT interval measurements of the source clinical studies were not performed on a consecutive beat-to-beat basis. The combination of both QT and RR histories in the same model also does not allow for a clear separation between QT/RR adaptation steepness and speed of QT/RR hysteresis.

Estimates of QT/RR hysteresis in the data derived from long-term ECG recordings are based on the assumption that the profile of the hysteresis (i.e., influence of the sequence of RR intervals preceding the measured QT interval) remains the same throughout the recordings. Such an assumption is an obvious simplification. Following rhythm abnormalities including supraventricular abnormalities, e.g., compensatory pauses of atrial ectopic beats, noticeable sinus pauses, abrupt vagal withdrawal, etc., noticeable QT interval changes might be detected on a beat-to-beat basis (the term “immediate effect” is occasionally used). In other situations, e.g., marked respiratory and other physiologic sinus arrhythmia, little or no beat-to-beat effects of heart period changes on the subsequent QT interval can frequently be detected (as in the example in Figure 1). It is therefore appropriate to study QT/RR hysteresis under different conditions, which led to our differentiation between heart rate acceleration and deceleration, which might be expected to reflect different sympatho-vagal reactions.

Our observation of, on average, slower hysteresis speed during heart rate acceleration contradicts a recent report by Axelsson et al. (2021) who observed the opposite. Nevertheless, they investigated the phenomenon in patients with permanent pacemakers and, thus, abnormalities in cardiac electrophysiology in whom QT/RR hysteresis might be different to that in the healthy subjects who were investigated in this study. The observations that we report were also based on heart rate changes due to physiologic reactions during drug-free days of pharmacologic investigations (i.e., due to movement and other responses to the environment in clinical research laboratories). Such situations might lead to electrophysiologic processes very different from responses to constant atrial or ventricular pacing at different fixed frequencies.

Initial studies on QT/RR hysteresis characterised the phenomenon by measuring the differences between QT intervals at the same instantaneous heart rate reached during rate acceleration (i.e., when QT interval duration is still influenced by slower heart rate in the past and, thus, is longer than it would correspond to the instantaneous heart rate under fully stabilised conditions) and deceleration (i.e., when QT interval duration is still influenced by previously faster rate and, thus, is shorter than the instantaneous heart rate would indicate) (Watanabe, 2007; Pelchovitz et al., 2012). These approaches led to measurements of the area of the “hysteresis window,” which was also used to study the influence of autonomic status and reflexes. Nevertheless, it is easy to understand that the measurements of hysteresis window area are highly problematic, since they depend on the speed of heart rate change (the faster the acceleration or deceleration of the rate, the greater the difference between the instantaneous rate and the rate derived from the history profile that influences QT duration). The reports of autonomic influences on the area of hysteresis window are consequently very difficult to interpret, since the speed of heart rate change (e.g., during and after physical exercise) is also subject to autonomic influence.

Because of these problems, the previous observations of autonomic influence on hysteresis window are difficult to compare with our observations that hysteresis speed declines with age, and that females show statistically faster hysteresis speed. These results might be interpreted as manifestations of cardiac autonomic status. Indeed, cardiac autonomic modulations are well-known to decrease with advancing age, and females have been shown to exhibit higher baseline parasympathetic modulations than males (Huikuri et al., 1996; Kuch et al., 2001; Hnatkova et al., 2019b). Thus, while QT/RR hysteresis might be influenced by cardiac autonomic status, other possibilities also exist that might explain our observations. These include a gradual buildup of myocardial fibrosis and an age-related increase in subclinical cardiac risk factors that might be expected to be more pronounced in males than in females.

### QT/QTc Variability

Although correcting QT interval duration for heart rate (or corresponding RR interval) derived from QT/RR hysteresis assessment leads to a substantial reduction in QTc variability (Malik et al., 2008a; Jacquemet et al., 2011, 2014; Hnatkova and Malik, 2020), the variability is not entirely eliminated as also demonstrated by the positive (i.e., > 0) regression residuals that we report (also note that the vertical width of the scatter diagram shown in Figure 15 spans some 10 ms). There are different sources of such a variability. In addition to long-term QTc changes [e.g., due to postprandial effects (Hnatkova et al., 2014; Taubel et al., 2014)], the short-term beat-to-beat variability of QT interval duration (Baumert et al., 2016) also play a role, since this QT instability cannot frequently be explained by underlying cardiac cycle variations. The details of the properties of short-term QT variability (Baumert et al., 2008; Malik, 2008; El-Hamad et al., 2015) and of the underlying mechanisms of repolarisation instability (Kenttä et al., 2011; Bauer et al., 2019) are beyond the scope of this text, but it needs to be noted that, because of rate independence, this variability cannot be addressed by correction of QT/RR hysteresis. This also explain the frequent absence (or even occasional reversal) of the so-called immediate effect of each RR interval, as we have already discussed in the previous section.

### Open Questions

Some studies suggested QT/RR hysteresis models that include a fixed immediate effect coefficient (Halámek et al., 2007), i.e., assume that the RR interval immediately preceding the QT interval measurement has always a substantially larger influence than the preceding RR intervals. As already discussed, such larger influence does not correspond to many observations, since the reproducibility of the immediate effect might be questioned. Nevertheless, it is also possible that hysteresis profiles differ under different physiologic circumstances. The duration of the subsequent QT interval might, thus, respond differently to atrial premature contraction than to a sinus nodal cycle with the same coupling interval. In this sense, atrial and ventricular pacing experiments and/or pharmacologically induced rapid heart rate changes (e.g., those by intravenous atropine or phenylephrine) might not be fully relevant to study the mechanisms that determine QT/RR hysteresis during physiologically regulated sinus rhythm. Rather, different physiologic reflexes leading to heart rate changes might need to be compared, e.g., heart rate changes during postural manoeuvres, head-up tilt, or mental stress testing.

Studies on specific clinical populations might provide further insight into the mechanisms of QT/RR hysteresis as well as into processes that influence it, especially since differences in hysteresis profiles have previously been linked to the difference in survival of cardiac patients. Little is presently known about QT/RR hysteresis in patients with abnormalities in autonomic and other cardiac regulation, e.g., patients with diabetic neuropathy, end-stage renal disease, thyroid dysfunction, or epilepsy. The role of cardiac structure would be better understood if QT/RR hysteresis was studied on patients with cardiac sarcoidosis, amyloidosis, lipomatosis, and cellular transplant rejection.

Presently, it is not known whether the sex difference in hysteresis speed occurs, similar to the differences in the individually corrected QTc intervals, around puberty (Andršová et al., 2019). Focused extensions of previous investigations in children and adolescents are needed to address this question.

### Additional Findings

In addition to the findings related to QT/RR hysteresis, our study also led to further observations. Consistent with previous reports (Malik, 2018), the data of the study show that the somewhat popular intra-subject QT/RR regression modelling based on logarithm transformation (i.e., the modelling that leads to individually corrected QTc intervals in the form QT/RR^α^) is less accurate than simple linear regressions. While the differences in the residuals of the QT/RR models are numerically small, they still play a role in power sample calculations of studies that depend on accurate QTc estimates (Malik et al., 2004).

QTc interval increases related to advancing age have previously been described (Rautaharju et al., 2014; Linde et al., 2018). Our study thus contributes only the observation that these age-related increases are also present when considering individually corrected QTc intervals rather than QTc values based on less accurate universal (i.e., not subject-specific) rate corrections. Somewhat surprisingly, we have not observed age influence on mean heart rate. However, this might have been affected by the calculation of the means across heart rates derived from the episodes when QT interval was measured rather than across the complete profile of the source Holter recordings.

The study data also agree with published observations that the slope of QT/RR adaptation is steeper in females than in males (Linde et al., 2018), which means that the marked sex difference in QT intervals observed at slower heart rates gradually diminishes as the heart rate increases. Other characters of the data [e.g., sex differences in the QT/RR curvatures (Malik et al., 2013)] also agree with existing reports, giving credibility to the analysed data collection.

### Limitations

The use of the exponential decay model of QT/RR hysteresis is the main limitation of the study. While it might be argued that the regression residuals are numerically so small that any improvement of the model could only lead to miniscule differences, we cannot be sure whether other hysteresis models would lead to the same results regarding the time-based and interval-based comparisons, sex differences in hysteresis speed, and differentiation between heart rate acceleration and deceleration. Since the study investigated Holter recordings of healthy subjects, we cannot comment on corresponding aspects in other well-defined clinical populations. The age range of the investigated subjects was limited; the population included only three subjects older than 60 years old. It is possible that with broader age ranges, other indices would also be found significantly age-dependent. Finally, we have not considered other data, such as body mass index, cognition function, or physical fitness characteristics, that might all also influence the investigated indices. The equations of linear regression analysis are based on the assumptions that independent and dependent variables (RR interval expressions and QT interval durations in this study) and the residual errors are normally distributed. This has not been tested in the study data; the test would be needed for each subject and each regression model separately (since all the regressions were performed for each subject separately). However, the number of ECG readings in each subject was large, thus minimising the effects of potential (albeit unlikely) departures from normal distributions (Schmidt and Finan, 2018). Also, normality tests have not been performed in the vast majority of studies investigating QT/RR regressions (Davey et al., 2000; Maury et al., 2013; Robyns et al., 2017; Yodogawa et al., 2021).

## Conclusion

The study provides further details on the physiology of QT/RR hysteresis. Analyses of the closeness of fit of the regression models between QT interval durations and RR intervals representing the underlying heart rate suggest that the response profile of QT/RR hysteresis appears to be driven by absolute time rather than the number of cardiac cycles over which the QT interval reacts to heart rate changes. We also observed that the speed of QT/RR hysteresis, contrary to the duration of individually rate-corrected QTc intervals, is decreased with increase in age. The slope of this increase was significantly steeper in females and thus, sex difference in hysteresis speed (which was faster in females) increases with age. In majority of the subjects, hysteresis speed was slower after heart rate acceleration than deceleration. The study also supported previous suggestions that the regression modelling between the QT and RR interval durations should not be based on logarithmic transformation (which has been popular in some studies on subject-specific QT heart rate corrections), since this transformation leads to poorer regression fit of data.

## Data Availability Statement

The raw data supporting the conclusions of this article will be made available by the authors, without undue reservation but pending the approval by the sponsors of the source clinical studies.

## Ethics Statement

The studies involving human participants were reviewed and approved by Focus in Neuss California Clinical Trials in Glendale Parexel in Baltimore Parexel in Bloemfontein PPD in Austin Spaulding in Milwaukee. The patients/participants provided their written informed consent to participate in this study.

## Author Contributions

IA, KH, and MM: study design and initial manuscript draft. KH and MM: software development and statistics and figures. GS, IA, KMH, MŠ, OT, PB, PS, and TN: ECG interpretation and ECG measurement. GS, TN, OT, and PS: supervision of the measurements. GS, MM, and TN: quality control of the measurements. GS, IA, KH, KMH, MM, MŠ, OT, PB, PS, and TN: final manuscript and approval of submission. All authors contributed to the article and approved the submitted version.

## Funding

This study was supported in part by the Ministry of Health, Czech Republic, conceptual development of research organization (Grant FNBr/65269705), by the British Heart Foundation New Horizons Grant NH/16/2/32499, and by the Specific Research of Masaryk University MUNI/A/1437/2020.

## Conflict of Interest

The authors declare that the research was conducted in the absence of any commercial or financial relationships that could be construed as a potential conflict of interest.

## Publisher's Note

All claims expressed in this article are solely those of the authors and do not necessarily represent those of their affiliated organizations, or those of the publisher, the editors and the reviewers. Any product that may be evaluated in this article, or claim that may be made by its manufacturer, is not guaranteed or endorsed by the publisher.
